# Cervical isometric strength and range of motion of elite rugby union players: a cohort study

**DOI:** 10.1186/2052-1847-6-32

**Published:** 2014-07-31

**Authors:** David F Hamilton, Don Gatherer

**Affiliations:** 1 Department of Trauma and Orthopaedics, University of Edinburgh, Chancellor’s Building, 49 Little France Crescent, Edinburgh EH164SB, UK; 2 Gatherer (Physiotherapy Limited), Tudor House, Aylesbury, UK

**Keywords:** Rugby, Cervical spine, Strength, Range of motion

## Abstract

**Background:**

Head and neck injury is relatively common in Rugby Union. Despite this, strength and range-of-motion characteristics of the cervical spine are poorly characterised. The aim of this study was to provide data on the strength and range-of-motion of the cervical spine of professional rugby players to guide clinical rehabilitation.

**Methods:**

A cohort study was performed evaluating 27 players from a single UK professional rugby club. Cervical isometric strength and range-of-motion were assessed in 3 planes of reference. Anthropometric data was collected and multivariate regression modelling performed with a view to predicting cervical isometric strength.

**Results:**

Largest forces were generated in extension, with broadly equal isometric side flexion forces at around 90% of extension values. The forwards generated significantly more force than the backline in all parameters bar flexion. The forwards had substantially reduced cervical range-of-motion and larger body mass, with differences observed in height, weight, neck circumference and chest circumference (p < 0.002). Neck circumference was the sole predictor of isometric extension (adjusted R^2^ = 30.34).

**Conclusion:**

Rehabilitative training programs aim to restore individuals to pre-injury status. This work provides reference ranges for the strength and range of motion of the cervical spine of current elite level rugby players.

## Background

Rugby union’s move to professionalism over recent years has markedly altered the physiques of elite level players; Dramatic increases in body mass, strength and power [1-3] has resulted in larger magnitude impact forces in the contact phases of the game. This has perhaps been driven by the strong association seen between teams containing the largest players and success in the professional game [1]. Alongside this change in player morphology, the number of contact events occurring during a match has risen four-fold [4]. Perhaps unsurprisingly, rugby union carries a comparatively high risk injury when compared with other sports [5,6].

Tackle based sports such as rugby exposes the cervical spine to potentially injurious forces, which are moderated by the musculoskeletal tissues [7]. While the relatively few catastrophic cervical injuries garner the most research and media interest, it has been suggested that the cumulative effects of these continuous shear and compression forces can have great impact on range of motion [8], muscle function [9] and proprioception [8,9] of the cervical spine. This may impair the spinal reflexes that act to stabilise and protect this vulnerable region, perhaps predisposing to further injury.

Head and neck injuries account for around 30% of all rugby injury events [6,10,11]. Considering that such injuries may involve multiple scans and clinical assessments, a substantial proportion of medical team input is devoted to treating and rehabilitating players to pre-injury status. It is surprising then that ‘pre-injury’ strength and range of motion characteristics of the cervical spine in professional rugby players has gone largely unreported in the literature. Conditioning of the cervical musculature has been proposed as a sensible (though unproven) method of mitigating neck injury [12,13], while post-injury muscle strength training is also a routinely employed rehabilitative tool, and is a primary treatment modality for neck pain [14]. Appropriate therapy intervention and muscle training loads cannot be applied without reference to expected maximal loading, with inadequate training loads clearly resulting in incomplete rehabilitation.

The primary aim of this study was to provide data on typical isometric strength parameters and range of motion of the cervical spine of current professional rugby players to guide clinical rehabilitation. Our secondary aim was to model factors predictive of cervical strength to aid clinical interpretation of recovery in the absence of individual specific muscle strength data.

## Methods

### Study design and population

This study was a retrospective evaluation of existing performance data. Assessment of cervical range of motion and isometric muscle strength was performed at a Welsh professional rugby union club on a single occasion (in 2011). Testing was carried out by the same individual. All players contracted to the club were invited to participate, providing they were free from head, neck or upper body injury. Three players were excluded with injury; five were not present at the testing session. No available players declined participation; 27 players consented to testing. Ethical approval was sought for this study form the South East Scotland REC; however the opinion was that this was retrospective evaluation of blinded data and did not require specific approval as the players originally consented to testing and the club consented to release blinded data for the purpose of evaluation.

### Assessments

Anthropometric measures were made of player height (Leicester Height Measure; SECA, UK), weight (medical grade mechanical flat scales; SECA, UK), neck circumference and chest circumference (standard fabric tape measure). Neck girth was assessed with the head in the anatomical position, using the thyroid cartilage as a reference position. Care was taken to tightly fasten the fabric measure around the neck but avoiding compression of the underlying tissues. Chest girth was assessed at full expiration, measuring the upper chest on the level of junction between the deltopectoral groove and tip of anterior axillary fold.

Cervical range of motion was assessed in three planes of reference (sagittal, frontal and transverse) using the Cervical Range of Motion Instrument (Performance Attainment Associates, Minnesota, USA) which has been thoroughly validated in various populations [15-18]. The assessment followed the test protocol advocated by Lark and McCarthy (2007), which has been previously described in detail [8]. Briefly, the participant was seated on a static chair with adjustable height so that the hips, knee and feet were positioned at 90°, and the head placed in the neutral anatomic position. An initial warm-up and familiarisation session practicing all movements to be assessed (flexion, extension, lateral flexion and rotation) was performed. Upon instruction, the participants moved their heads in the test parameter (looking directly ahead) and held the end of range position to allow the assessor to record the angle achieved, following which, the individual returned their head to the neutral position. Three separate measures were made in each direction, the mean of which is reported.

### Neck strength assessment

Isometric cervical muscle testing is well validated [19-21]. We assessed maximal voluntary isometric cervical muscle strength in three planes of reference with the GS Gatherer and GS Analysis Suite (Gatherer Systems Ltd, Aylesbury); a custom-built device based on a 300 Kg load cell and bespoke software system. The test was performed employing a previously reported protocol [22], specifically validated in young rugby players [13], subjecting the neck to manually controlled linear incremental loading to test positional failure in the absence of pain or neurological symptoms (which stopped the test). The head was held in the neutral anatomic position at all times throughout the test. Peak isometric force was logged at the point of head movement with loss of test position. Loading was applied and data were recorded at 20Hz. Peak isometric force generated by the musculature was defined as the maximal load recorded during the test procedure. An average of 3 tests is reported.

### Statistical analysis

Data were assessed for normality and are reported as means with standard deviations as a measure of dispersion. Independent samples t-tests were used to assess differences in continuous variables between groups unless otherwise stated. Significance was accepted as p < 0.05 incorporating the Benjami-Hochberg FDR correction [23,24] for the testing of multiple hypotheses, to reduce the possibility of making a type II error in the interpretation of results. Pearson correlation coefficients are reported for bivariate correlations. Multivariate stepwise regression modelling was performed to achieve the most predictive model utilising the fewest variables. Predictive variables were entered into the model using an alpha of p < 0.1 to accommodate the possibility of variables achieving statistical significance once the confounding influence of additional variables was controlled. All analysis was carried out using Minitab (release 16) software.

## Results

**Figure 1:**
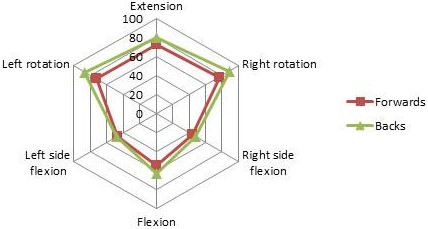
Radar graph demonstrating the pattern of range of motion of the cervical spine of elite rugby players, highlighting differences between forwards and backs.

Descriptive data of the anthropometric variables assessed are presented in Table 1. The mean age of the players was 23 years, with the forwards on average 2 years older than the backs. As expected, the forwards had a substantially larger body mass than the back line, with highly significant differences observed in height, weight and measures of neck and chest circumference (p < 0.002). The forwards presented with a notably reduced cervical range of motion compared to the backline, with significant differences of around 10 degrees observed in measures of flexion and rotation (Table 1, Figure 1).

**Table 1:** Anthropometric data

	**All players**	**Forwards**	**Backs**	**Significance**
n	27	16	11	0.317*
Age (years)	22.95 (3.94)	26.00 (4.43)	23.70 (2.98)	0.164
Height (cm)	186.21 (6.40)	191.00 (6.25)	182.50 (3.87)	0.002^‡^
Weight (kg)	97.94 (10.73)	107.42 (5.47)	89.57 (6.01)	<0.001^‡^
Neck girth (cm)	41.90 (2.65)	43.41 (1.96)	39.55 (1.50)	<0.001^‡^
Chest girth (cm)	105.62 (5.49)	107.72 (4.90)	101.70 (4.37)	<0.001^‡^
CROM (degrees)				
Flexion	57.92 (9.37)	54.38 (7.94)	63.20 (8.79)	0.019^‡^
Extension	75.48 (11.84)	72.06 (12.97)	79.40 (9.05)	0.103
Left side flexion	48.84 (8.48)	47.38 (9.26)	48.20 (7.08)	0.80
Right side flexion	45.20 (8.29)	43.88 (8.08)	47.60 (8.10)	0.268
Left rotation	78.40 (13.17)	72.75 (11.70)	87.20 (9.58)	0.002^‡^
Right rotation	81.32 (12.94)	76.75 (12.86)	88.70 (8.59)	0.009^‡^

All data reported as mean (SD). CROM = cervical range of motion. *Chi square. ^‡^Remains significant at p = 0.05 adjusting for multiple comparisons.

**Figure 2:**
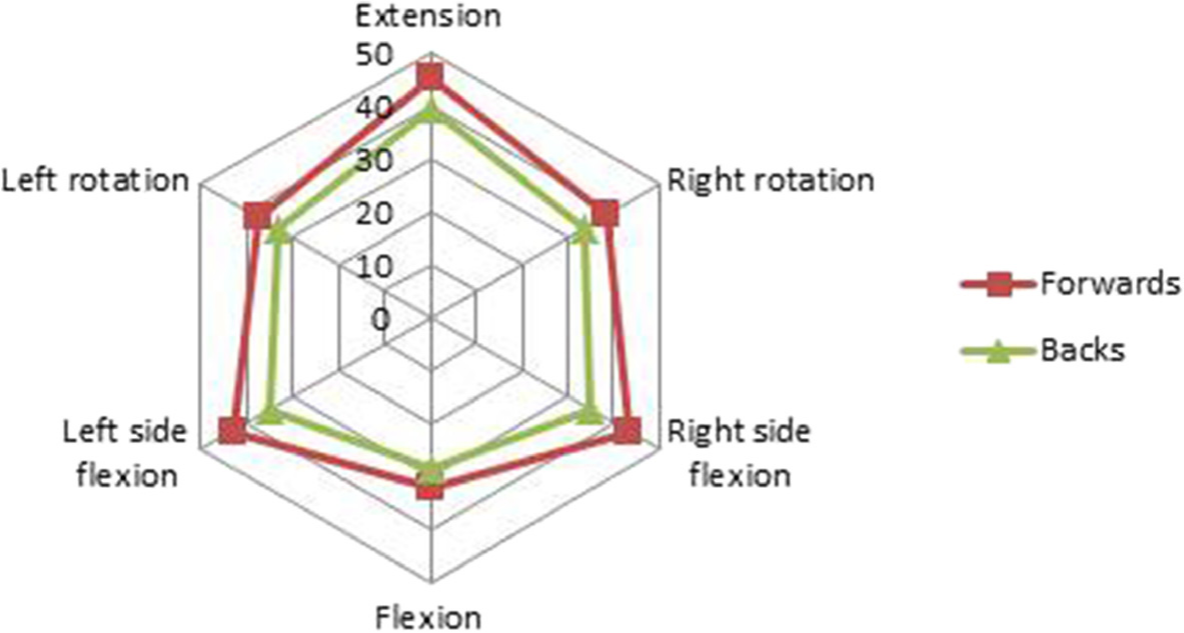
Radar graph demonstrating the pattern of isometric strength of the cervical spine of elite rugby players, highlighting differences between forwards and backs.

The largest isometric forces were generated in extension, with broadly equal isometric left and right side flexion forces at around 90% of peak extension values. The forwards generated significantly larger forces than the backline in all test parameters bar isometric flexion (Table 2, Figure 2). Proportional differences between flexion and extension forces were however consistent between player groups, with flexion 27.56% (SD 12.02%) reduced compared to extension in the forwards and 27.50% (13.85%) in the backs. Differences between left and right side flexion forces were similar between forwards and backs [7.19% (7.61%) and 8.40% (7.34%) respectively], as were differences between left and right isometric rotation [6.19% (4.61%) and 6.50% (3.89%) in the forwards and backs respectively.

**Table 2:** Cervical isometric neck strength (kg)

	**All players**	**Forwards**	**Backs**	**Significance**
Flexion	30.92 (5.20)	32.00 (5.60)	28.54 (3.98)	0.079
Extension	43.39 (6.39)	44.92 (7.12)	39.52 (5.12)	0.035^‡^
Left side flexion	40.54 (6.97)	42.90 (7.70)	35.02 (4.50)	0.003^‡^
Right side flexion	40.34 (7.60)	43.15 (7.58)	35.03 (4.53)	0.002^‡^
Left rotation	36.09 (6.15)	37.56 (6.96)	33.08 (3.31)	0.006^‡^
Right rotation	36.69 (5.38)	38.55 (5.52)	33.46 (3.04)	0.038^‡^

All data reported as mean (SD). ^‡^Remains significant at p = 0.05 adjusting for multiple comparisons.

### Relationship between neck strength and anthropologic variables

**Figure 3:**
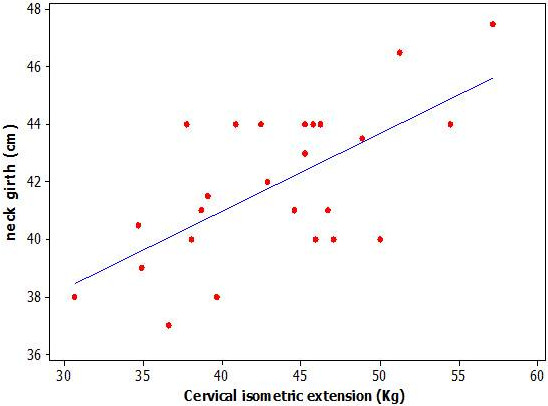
**Relationship between global neck strength and neck circumference (R**^**2**^ **= 30.34).**

A strong association was observed between isometric cervical extension and neck circumference (r = 0.65), though not with chest circumference (r = 0.27). Modest association was observed with body weight (r = 0.49). Cervical range of motion in flexion and rotation was modestly associated with isometric extension (r = 0.54 and 0.40 respectively), extension and side flexion were poorly associated (Table 3). Multivariate stepwise regression modelling of global isometric neck strength revealed neck circumference to be the sole predictor of isometric extension, and accounted for around a third of the variation in cervical isometric extension (adjusted R^2^ = 30.34) (Figure 3).

**Table 3:** Bivariate analysis of predictors of global isometric neck strength

**Predictor variable**	**r**	**Significance**
Age	0.27	0.224
Weight	0.49	0.034
Height	0.27	0.267
Neck girth	0.65	0.001
Chest girth	0.26	0.205
CROM flexion	−0.54	0.005
CROM extension	−0.15	0.471
CROM left side flexion	−0.10	0.645
CROM right side flexion	−0.13	0.545
CROM left rotation	−0.38	0.059
CROM right rotation	−0.40	0.049

## Discussion

This study presents baseline data for the cervical isometric strength and range of motion of UK based professional rugby players. Generally high maximal strength values were observed, with significant differences evident between the forwards and backline players. The forwards were bigger and heavier with correspondingly larger neck circumference. These factors were related to both maximal strength and range of motion; the forwards displaying notably reduced flexion and rotation. Interestingly however in multivariate modelling accounting for confounding variables, only the neck circumference was predictive of maximal isometric strength.

While we would expect strength values of professional athletes to be substantially higher than that of the general population, the values presented here are three times greater than those reported in a healthy Danish population. Jordan et al. (1999) reported values of around 14 Kg for isometric extension for 20–30 year old males [20]. This though was a small cohort of a larger population (n = 10) and these individuals weighed only 80 Kg, some 20 Kg lighter than the professional rugby players assessed in this study. More recently, Salo et al. (2006) reported the isometric neck strength of healthy females [25], finding the isometric extension strength of similarly aged women to be around 20 Kg, seemingly in proportion to our findings. Again these individuals were markedly smaller than this group at around 167 cm in height and 62 kg in weight.

Various equipment and testing protocols have been used in different studies of cervical isometric strength making it difficult to compare findings. A particular problem in comparing to wider literature is the propensity to use isokinetic dynamometers to assess an isometric load. These results are typically presented as torques, though the length of the moment arm employed is rarely defined to allow proper evaluation of the torque values presented. Generally it must be assumed that the moment arm used in these studies relates to the position of the arm of the testing machine relative to its rotational axis. This methodology fails to account however for the length of the individuals’ neck, which need also be taken into consideration as this clearly influences the actual torque generated. The only directly comparable data available (using the same testing regime and equipment) is a large cross-sectional study of rugby playing Scottish schoolchildren [13]. In this cohort, increasing values were observed between the ages of 12 and 18 with mean isometric extension values ranging between 20 and 30 Kgs, again seemingly consistent with this report of professional athletes. The ratio of flexion to extension strength of around 70% presented here is however consistent across previous reports [12,19,20,25] lending validity to these results.

An intuitive relationship between isometric neck strength and body mass is typically reported, though the strength of this association is poorly defined, perhaps partly due to the difficulty noted in comparing studies and also perhaps that previous studies have assessed various healthy and clinical populations. In these elite rugby players, though increased player weight and reduced cervical rotation were certainly associated with stronger necks, the only relevant predictor in our modelling was neck circumference. It has been previously shown that neck muscle cross-sectional area is related to muscle strength [26]. Interestingly, in a previous assessment of adolescent rugby players we found that neck circumference was not associated with cervical strength, but that a regression algorithm using player age, weight and grip strength was strongly predictive of isometric extension [13]. In the physically mature rugby players assessed here, a comparatively strong association is observed; highlighting that once fully developed, changes in neck circumference are likely to reflect the muscle volume of the individual. This larger muscle mass is also likely to be associated with the reduced flexion and rotation seen in the forward players.

The primary limitation of this study is the comparatively small sample size and therefore the wider generalisiblity. The number of participants is consistent with most other work in this area and this limitation is mitigated by assessing all available players in a representative elite rugby club. This group is likely to represent a relatively homogeneous population of highly trained individuals reflective of their peers. The values reported here are notably higher than those seen in untrained populations, and in younger players, as would be expected. It must also be noted that this assessment was a single point in time, and we cannot therefore comment as to whether strength and range of motion parameters change over the season. It is reasonable to suggest that enhanced strength and flexibility are developed with training interventions over the season and (as with the wider musculoskeletal system) particular attention should be paid in the pre-season conditioning phase to mitigate injury. Further work is however needed to confirm this suggestion.

## Conclusion

Rehabilitative training programs typically aim to restore the individual to pre injury status. This work provides reference ranges for the strength and range of movement of the cervical spine of current elite level UK based rugby players. In this group, neck strength is most associated with neck circumference, which reflects the underlying cross-sectional muscle area, though the predictive value of this parameter was comparatively low, explaining around a third of the variation in strength. Ultimately, as with any rehabilitative intervention, specific ‘pre-injury’ parameters of each individual are beneficial to appropriately guide rehabilitation and conditioning programmes.

## Competing interests

DG has shares in Gatherer Systems which provided the cervical test equipment for this study. DH declares no competing interests.

## Authors’ contributions

Both authors designed the study, DG collected the data, DH analysed the data, DH drafted the manuscript, both authors revised and approved the final submission.

## Pre-publication history

The pre-publication history for this paper can be accessed here:

http://www.biomedcentral.com/2052-1847/6/32/prepub

## References

[B1] Sedeaud A Marc A Schipman J Tafflet M Hager JP Toussaint JF How they won Rugby World Cup through height, mass and collective experience Br J Sports Med 2012 46 580 22348873 10.1136/bjsports-2011-090506

[B2] Murray AD Murray IR Robson J Rugby Union: faster, higher, stronger: keeping an evolving sport safe Br J Sports Med 2012 0 1 2 doi:10.1136/bjsports-2012-091844 10.1136/bjsports-2012-091844 23264556

[B3] Duthie G Pyne D Hooper S Applied physiology and game analysis of rugby union Sports Med 2003 33 973 991 14606925 10.2165/00007256-200333130-00003

[B4] Quarrie KL Hopkins WG Changes in player characteristics and match activities in Bledisloe Cup rugby union from 1972 to 2004 J Sports Sci 2007 25 895 903 17474043 10.1080/02640410600944659

[B5] Palmer-Green DS Stokes KA Fuller CW England M Kemp SP Trewartha G Match injuries in English youth academy and schools rugby union: an epidemiological study Am J Sports Med 2013 41 749 755 23380159 10.1177/0363546512473818

[B6] Brooks JHM Kemp SPT Injury-prevention priorities according to playing position in professional rugby union players Br J Sports Med 2011 45 765 775 20484316 10.1136/bjsm.2009.066985

[B7] Torg J Ramsey-Emrhein J Suggested management guidelines for participation in collision activities with congenital, developmental, or post injury lesions involving the cervical spine Med Sci Sports Exerc 1997 29 7 256 272 10.1097/00005768-199707001-00008 9247923

[B8] Lark SD McCarthy PW Cervical range of motion and proprioception in rugby players versus non-rugby players J Sports Sci 2007 25 887 894 17474042 10.1080/02640410600944543

[B9] Pinsault N Anxionnaz M Vuillerme N Cervical joint position sense in rugby players versus non-rugby players Phys Ther Sport 2010 11 66 70 20381004 10.1016/j.ptsp.2010.02.004

[B10] Bottini E Poggi EJ Luzuriaga F Secin FP Incidence and nature of the most common rugby injuries sustained in Argentina (1991–1997) Br J Sports Med 2000 34 94 97 10786863 10.1136/bjsm.34.2.94 PMC1724172

[B11] McIntosh AS McCrory P Finch CF Wolfe R Head, face and neck injury in youth rugby: incidence and risk factors Br J Sports Med 2010 44 188 193 18385188 10.1136/bjsm.2007.041400

[B12] Olivier PE Du Toit DE Isokinetic neck strength profile of senior elite rugby union players J Sci Med Sport 2008 11 96 105 17560830 10.1016/j.jsams.2007.01.009

[B13] Hamilton DF Gatherer D Jenkins PJ Maclean JGB Hutchison JD Nutton RW Simpson AHRW Age-related differences in the neck strength of adolescent rugby players: A cross-sectional cohort study of scottish schoolchildren Bone Joint Res 2012 1 152 157 23610685 10.1302/2046-3758.17.2000079 PMC3626274

[B14] Sarig-Bahat H Evidence for exercise therapy in mechanical neck disorders Man Ther 2003 8 10 20 12586557 10.1054/math.2002.0480

[B15] Tousignant M De Bellefeuille L O'Donoughue S Grahovac S Criterion validity of the cervical range of motion (CROM) goniometer for cervical flexion and extension Spine (Phila Pa 1976) 2000 25 324 330 10703104 10.1097/00007632-200002010-00011

[B16] Tousignant M Duclos E Laflèche S Mayer A Tousignant-Laflamme Y Brosseau L O'Sullivan JP Validity study for the cervical range of motion device used for lateral flexion in patients with neck pain Spine (Phila Pa 1976) 2002 27 812 817 11935102 10.1097/00007632-200204150-00007

[B17] Audette I Dumas JP Côté JN De Serres SJ Validity and between-day reliability of the cervical range of motion (CROM) device J Orthop Sports Phys Ther 2010 40 318 323 20436238 10.2519/jospt.2010.3180

[B18] Youdas JW Garrett TR Suman VJ Bogard CL Hallman HO Carey JR Normal range of motion of the cervical spine: an initial goniometric study Phys Ther 1992 72 770 780 1409874 10.1093/ptj/72.11.770

[B19] Garge´s GL Medina D Milutinovic L Garavote P Guerado E Normative database of isometric cervical strength in a healthy population Med Sci Sports Exerc 2002 33 464 470 10.1097/00005768-200203000-00013 11880811

[B20] Jordan A Mehlsen J Bu¨low PM Ostergaard K Danneskiold-Samsoe B Maximal isometric strength of the cervical musculature in 100 healthy volunteers Spine 1999 24 1343 1348 10404577 10.1097/00007632-199907010-00012

[B21] Chiu TT Sing KL Evaluation of cervical range of motion and isometric neck muscle strength: reliability and validity Clin Rehabil 2002 16 851 858 12501947 10.1191/0269215502cr550oa

[B22] Peek K Gatherer D The rehabilitation of a professional rugby union player following a C7/T1 posterior microdiscectomy Phys Ther Sport 2005 6 195 200

[B23] Benjamini Y Hochberg Y Controlling the false discovery rate: a practical and powerful approach to multiple testing J R Statist Soc B 1995 57 289 300

[B24] Storey JD "The positive false discovery rate: A Bayesian interpretation and the q-value" Ann Stat 2003 31 2013 2035

[B25] Salo PK Ylinen JJ Mälkiä EA Kautiainen H Häkkinen AH Isometric strength of the cervical flexor, extensor, and rotator muscles in 220 healthy females aged 20 to 59 years J Orthop Sports Phys Ther 2006 36 495 502 16881466 10.2519/jospt.2006.2122

[B26] Mayoux-Benhamou MA Wybier M Revel M Strength and cross-sectional area of the dorsal neck muscles Ergonomics 1989 32 513 518 2767044 10.1080/00140138908966121

